# Coexistence of anomalous muscle, persistent median artery, bifid median nerve causing carpal tunnel syndrome: A case report and literature review

**DOI:** 10.3389/fped.2023.1043442

**Published:** 2023-02-09

**Authors:** Jun Qin, Xia-xian Tan, Ming-qiang Xue, Jing-wei Wang, Jin-min Zhao, Ke Sha

**Affiliations:** ^1^ Department of Orthopaedics Trauma and Hand Surgery, The First Affiliated Hospital of Guangxi Medical University, Nanning, China; ^2^ Department of Medical Examination Center, Guilin People's hospital, Guilin, China

**Keywords:** juvenile carpal tunnel syndrome, abnormal lumbrical muscle, persistent median artery, bifid median nerves, treatment, case report

## Abstract

Carpal tunnel syndrome (CTS) is an upper extremity median nerve entrapment disorder that is rare in children and adolescents. Anatomical variations of the wrist, such as anomalous muscles, persistent median artery (PMA), and bifid median nerves (BMN), are rare etiology of CTS. Coexistence of all three variants combined with CTS in adolescents has been rarely reported. Case description: A 16-year-old right-hand dominant male presented to our clinic with several years of bilateral thenar muscle atrophy and weakness but no paresthesia or pain in his both hands. Ultrasonography showed that the right median nerve become significantly thinner, and the left median nerve was split into two branches by PMA. Magnetic resonance imaging (MRI) revealed that anomalous muscles in the bilateral wrist extending to the carpal tunnel, causing compression of the median nerve. Considering the possibility of CTS clinically, the patient underwent bilateral open carpal tunnel release without resection of anomalous muscles and PMA. The patient has no discomfort after 2 years. This suggests that anatomical variations of the carpal tunnel may contribute to CTS, which can be confirmed by preoperative ultrasonography and MRI, and the possibility of carpal tunnel anatomical variations should be considered when CTS occurs in adolescents. Open carpal tunnel release is an effective treatment for juvenile CTS without the need to resect abnormal muscle and PMA during the operation.

## Introduction

Carpal tunnel syndrome (CTS), the most common upper extremity nerve entrapment disease and mostly occur in adult women, is characterized by paresthesia and numbness in the area innervated by the distal median nerve, usually accompanied by atrophy and weakness of the thenar muscle. Compression of the median nerve at the wrist is the etiology of CTS, and the space-occupying lesions and anatomical variations of the wrist are the potential causes of CTS.

CTS in children and adolescents is rare and mostly secondary to various hereditary diseases, such as lysosomal storage disease, including mucopolysaccharidoses(MPS) and mucolipidoses (ML) (1). Furthermore, wrist anatomical variations, wrist trauma, and localized masses may also contribute to carpal tunnel syndrome (2). Although anatomical variations of the wrist are not uncommon in clinical, only some individuals experience symptoms. The incidence of anatomical variants in CTS is approximately 5.7%–8.9%, and the common variants are anomalous muscle (lumbrical muscles, flexor digitorum muscle, palmaris profundus, palmaris longus muscle, etc.), persistent median artery (PMA), and bifid median nerves (BMNs) (3, 4). Anomalous muscle occurs in isolation, while PMA often coexists with BMN, but the coexistence of the three variants combined with carpal tunnel syndrome is very rare.

We report a rare case of bilateral CTS in an adolescent, which presents anomalous muscle in the bilateral carpal tunnel, and coexistence of PMA and BMNs in the left carpal tunnel. Knowledge about the anatomic variations of carpal tunnel is necessary to avoid any iatrogenic injuries.

## Case description

**Figure 1:**
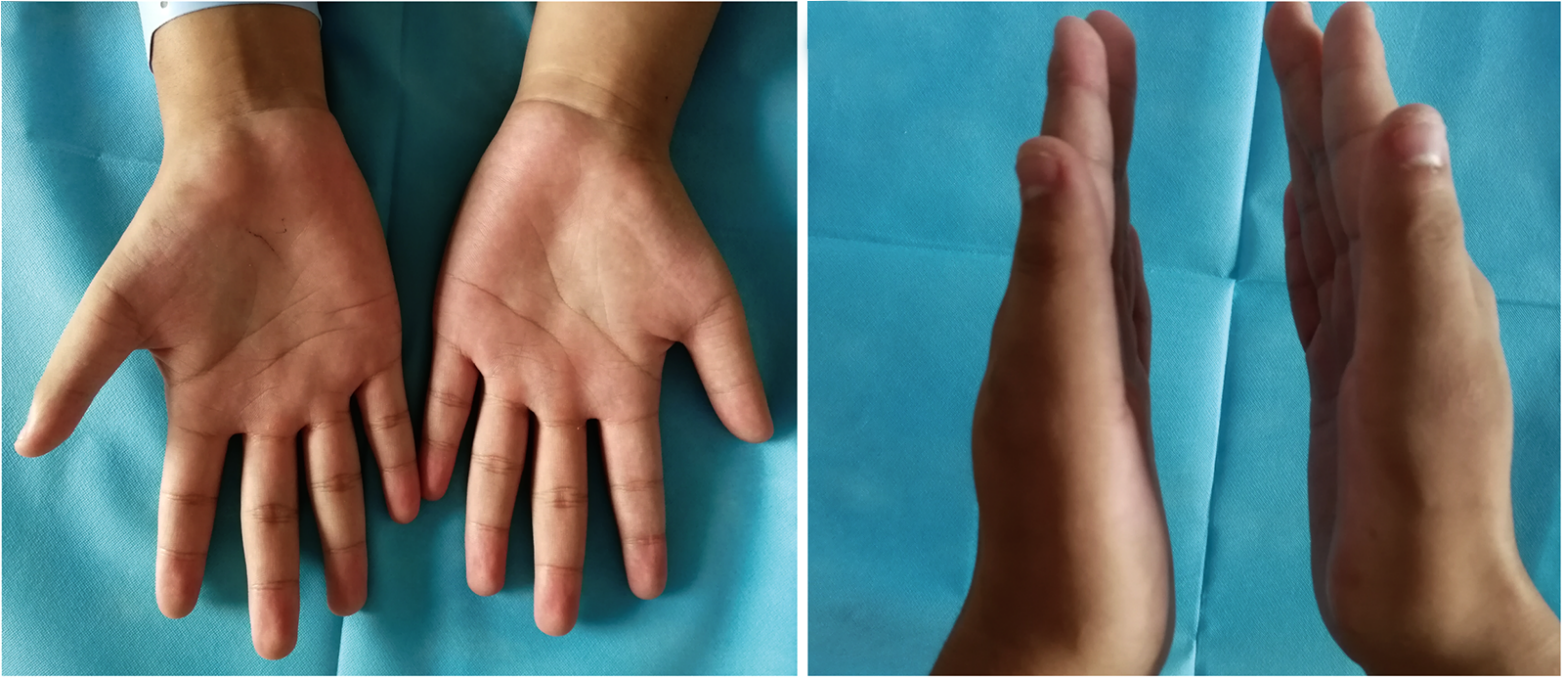
Preoperative physical examination. Bilateral thenar muscle atrophy, and the right thenar muscle was significantly atrophied compared to the contralateral side.

**Figure 2:**
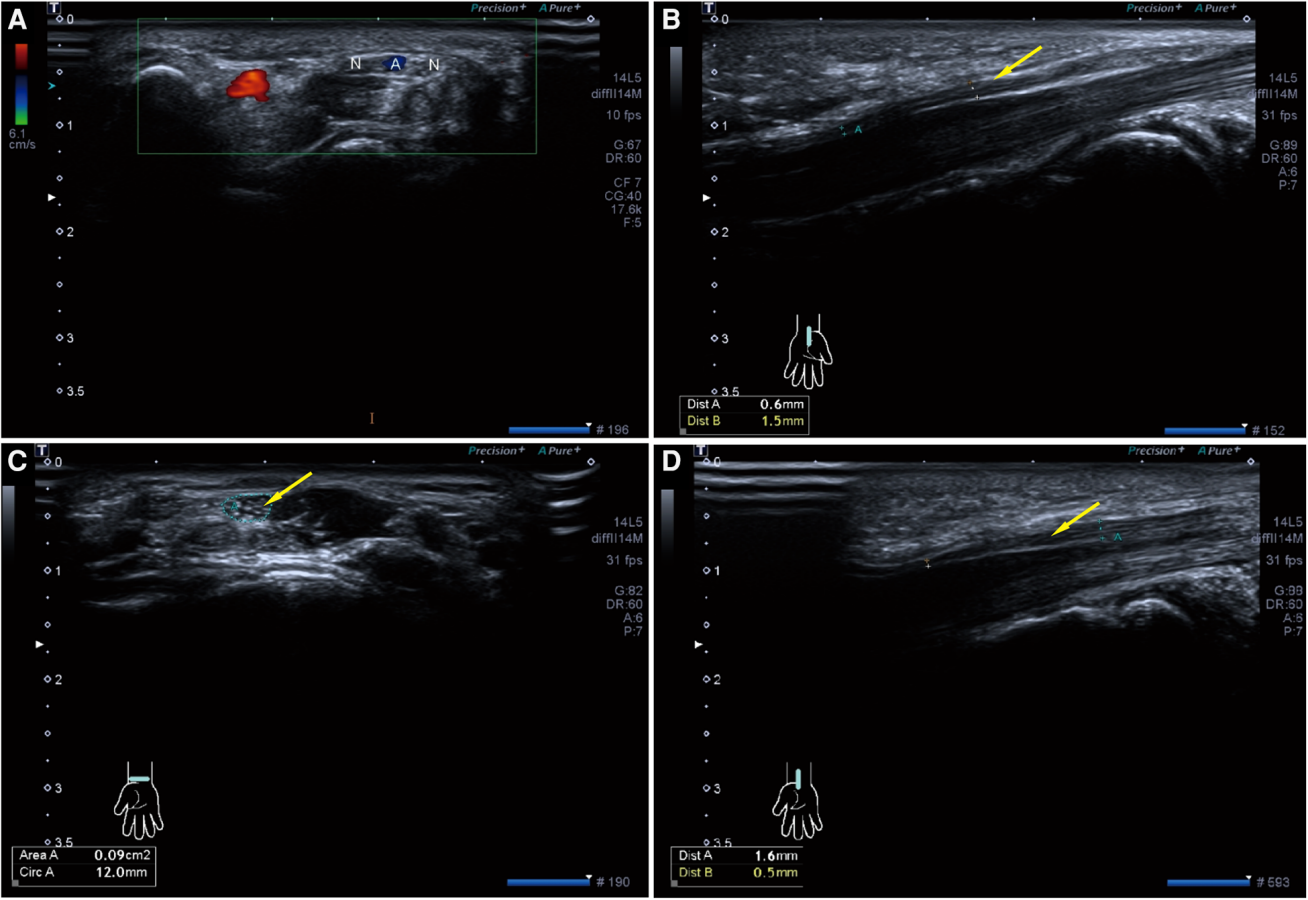
Preoperative high-resolution ultrasonography of both wrists. (**A**) Axial ultrasonography of left wrist shows bifid median nerve (N) with persistent median artery (**A,B**) Sagittal ultrasonography of left carpal tunnel shows the left median nerve (Yellow arrow) becomes significantly thinner after entering the carpal tunnel. (**C,D**) Axial and Sagittal ultrasonography of right carpal tunnel also shows the right median nerve (Yellow arrow) becomes significantly thinner after entering the carpal tunnel.

**Figure 3:**
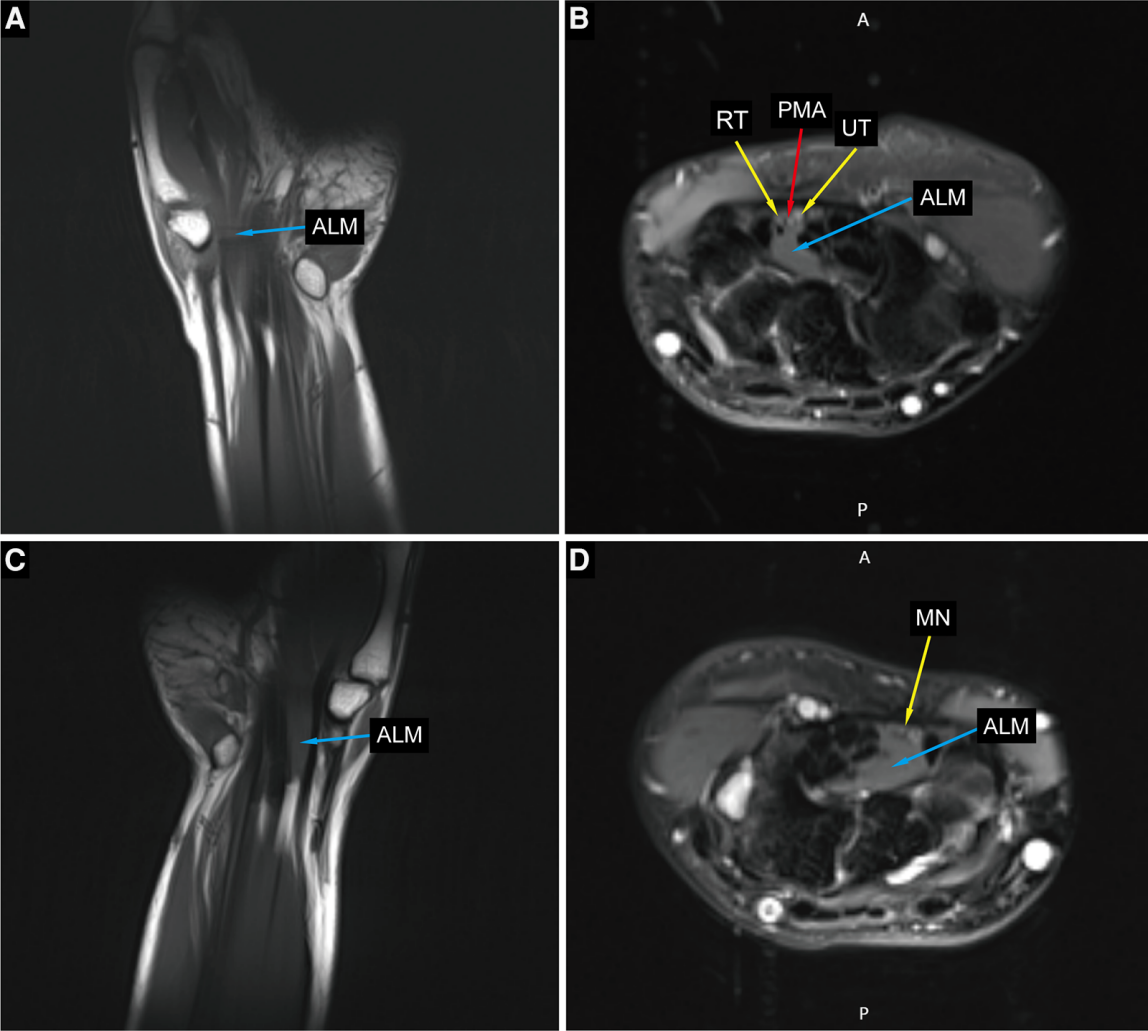
Preoperative MRI imaging of the bilateral hands and wrists. (**A,C**) Coronal sections of the left and right hand show an anomalous lumbrical muscle in carpal tunnel. (**B**) Axial section at left hamate hook level showing anomalous lumbrical muscle, bifid median nerve with persistent median artery. (**D**) Axial section at right hamate hook level showing anomalous lumbrical muscle and compressed median nerve. ALM, anomalous lumbrical muscle; RT, radial trunk of the median nerve; UT, ulnar trunk of the median nerve; PMA, persistant median artery; MN, median nerve.

A 16-year-old right-hand dominant male presented to our clinic with several years of bilateral thenar muscle atrophy and weakness. He reported no paresthesia or pain in his both hands, and no history of wrist trauma before the onset of symptoms. ­On examination, he had noticeable wasting of the thenar muscle in his right hand compared with the contralateral side, and the muscle strength of abductor pollicis brevis muscle and opponens pollicis muscle were slightly reduced to 5^−^/5 (MRC) (Figure 1). Phalen's test and Tinel's signs over median nerve were negative. No abnormalities were seen on the bilateral upper arm and forearm physical examination. As shown in Figure 2 and Table 1, high-resolution ultrasonography of both wrists was performed and showed that the right median diameter becomes significantly thinner after entering the carpal tunnel, and the left median nerve is split into two branches by PMA, which are compressed and flattened as they pass through the carpal tunnel. However, anomalous muscles within carpal tunnel were ignored. Magnetic resonance imaging (MRI) of the wrist revealed that bilateral adductor pollicis extended to carpal tunnel causing compression of the median nerve, and axial fat-suppressed PD-weighted images at level of hook of hamate showed segmental swelling of median nerve, while PMA was missed on MRI (Figure 3, Supplementary Videos S1, S2). As shown in Supplementary Table S1 and Supplementary Figure S1, the median nerves cross-sectional area(CSA) at different axial MRI levels showed that the right median CSA became smaller after entering the carpal tunnel, while the left median CSA became larger. Electrophysiologic testing suggested that the right median nerve compound muscle action potential (CMAP) amplitude was 2.9 mV proximally and 2.8 mV distally and was smaller when compared with the left median (13.8 mV proximally and 14.0 mV distally), while both medians had normal latency, conduction velocity, and sensory nerve action potentials(SNAP). Needle electromyography showed that the recruitment of motor unit potentials(MUP) in the right abductor pollicis brevis muscle was absent and the MUP duration was slightly prolonged, and the MUP amplitude was normal. Taken together, these results are suggestive of neurogenic damage.

**Table 1:** MN diameter and cross-sectional area in the carpal tunnel.

Nerve	MN diameter(mm)		MN cross-sectional area (mm^2^)		
	Proximal	Distal	radial-ulnar junction level	Pisiform level	Hook of the hamate level
RMN	1.6	0.5	11.88	9.27	7.42
LMN	1.5	0.6	7.32	8.63^a^	12.14^a^

RMN, the right median nerve; LMN, the left median nerve.

^a^ a sum of the radial trunk and ulnar trunk of the median nerve cross-sectional area.

**Figure 4:**
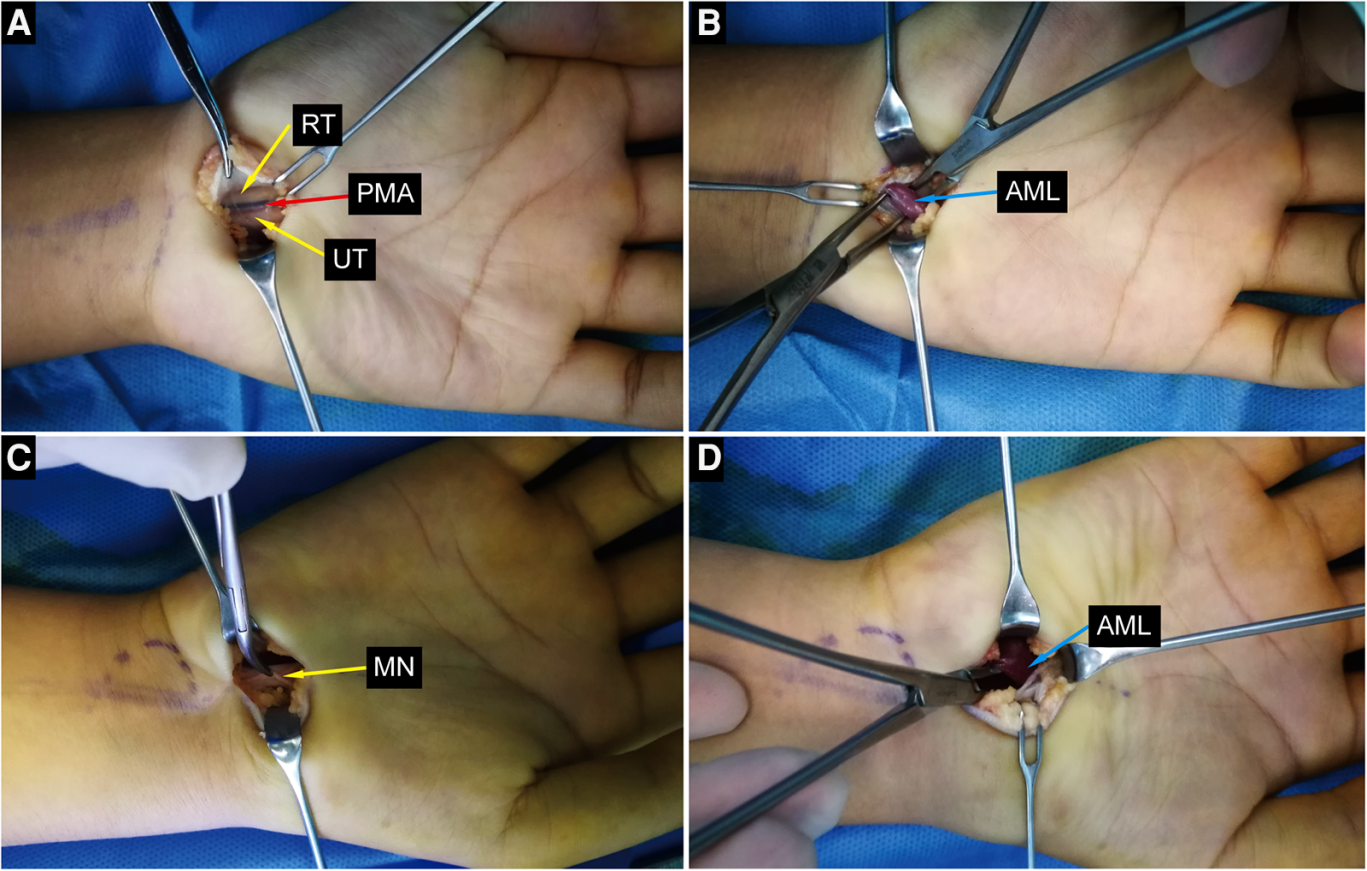
Intraoperative photographs. (**A,B**) Intraoperative photographs of the left wrist show bifid median nerve, persistent median artery and anomalou lumbrical muscle. (**C,D**) Intraoperative photographs of the right wrist show compressed median nerve and anomalou lumbrical muscle. ALM, anomalous lumbrical muscle; RT, radial trunk of the median nerve; UT, ulnar trunk of the median nerve; PMA, persistant median artery; MN, median nerve.

The possibility of CTS was considered clinically, and bilateral open carpal tunnel release was performed on the patient. Operative exploration was shown in Figure 4, the right median nerve was obviously compressed at the carpal tunnel, a thickened epineurium and a thinner nerve were seen, and the recurrent thenar branch of the median nerve was compressed by fibrous connective tissue. BMN was found in the left wrist, accompanied by PMA with a diameter of about 2.0 mm, the artery divides the median nerve into two branches, and the nerves are locally compressed and flattened. Anomalous muscles were found in the bilateral carpal tunnel and below the course of the median nerve. When the fingers were passively straightened, the muscles could still be seen in the carpal tunnel. When the muscles were stretched, metacarpophalangeal joints of index fingers were passively flexed and interphalangeal joints were passively straightened, lumbrical anatomical variation was considered. During the operation, transverse carpal ligaments were incised and the median nerves were carefully released to the recurrent branch of thenar muscles, the anomalous muscles and the PMA were not removed but were left *in situ*. Routine dressing changes were performed after surgery, and active and passive movements of wrist joints and fingers were performed early to reduce hand edema and local adhesion. The surgical incision healed well and no complications were found.

Regular follow-up was performed post-operative and the last follow-up period was two years after the operation, with no complaints of discomfort. Physical examination showed that bilateral thenar muscles still atrophied, but the atrophy was not further aggravated compared with two years ago (Supplementary Figure S2). The strength of the bilateral muscle abductor pollicis brevis muscle and opponens pollicis muscle were 5^−^/5 (MRC).

## Discussion

CTS is the most common clinical upper extremity nerve entrapment disease, which is characterized by paresthesia and motor dysfunction of the median nerve innervation due to the entrapment of the median nerve when passing through the carpal tunnel. Common causes of CTS are menopause, diabetes, hypothyroidism, obesity, arthritis, and pregnancy (5), and other rare causes include carpal tunnel anatomical variation, gout, carpal tunnel space-occupying lesions, etc (6–8).. Although CTS is a very common disorder, CTS in adolescents is rare. The case we encountered did not have the typical clinical manifestations, but only thenar atrophy and weakness, so we were confused during the diagnosis. With the help of ultrasonography, MRI, and electromyography, abnormal anatomical structures were found in the carpal tunnel, thus confirming the diagnosis. Notably, preoperative ultrasonography failed to detect abnormal muscles, while MRI misidentified abnormal muscles as adductor pollicis muscles. However, the abnormal muscles were confirmed lumbrical muscles during intraoperative exploration, which was consistent with most studies, and the misjudgment may be due to insufficient awareness of abnormal muscles, which is similar to another case (6). Ultrasonography, MRI, and electromyography are commonly used as auxiliary examinations for CTS, when anatomical abnormalities in the carpal tunnel are suspected, those examinations may be required (9, 10).

Although rare in clinical practice, anatomical variations in the carpal tunnel, such as anomalous muscles, PMA, and BMN, may all contribute to the occurrence of CTS. Muscle variations in the carpal tunnel are mostly discovered accidentally during surgery, and some are unexpectedly detected during autopsy. The prevalence of anomalous muscles in CTS is about 1.3% to 2.9% (3, 11), while the true incidence of nerve entrapment due to muscle variants is unknown (12). The sources of anomalous muscles can be lumbrical muscle (13, 14), flexor digitorum superficialis (15), palmaris profundus (16), palmaris longus muscle (17), etc. and lumbrical muscle origin was the most common among them.

Several authors reported that they found abnormally lumbrical muscles, which origin extended proximally across the carpal tunnel, during open carpal tunnel release, and they suggest that aberrant lumbrical muscles contribute to the development of CTS (13, 18). Additionally, it has also been reported that trauma-induced hematoma of variant muscle within the carpal tunnel can lead to acute CTS (19). Cartwright et al. (20) showed that compared with participants without CTS, participants with CTS had more muscle within the carpal tunnel with their wrists in neutral and flexed positions. Interestingly, however, some aberrant muscles in the carpal tunnel can be clinically asymptomatic, and CTS occurs only in a small number of them (21). Cobb et al. (22) believed that the lumbrical muscle entering the carpal tunnel during finger flexion is a normal phenomenon, and the occurrence of CTS may be related to the nature of its work. Thus, muscle variation in the carpal tunnel is associated with CTS and is a risk factor for CTS in manual workers who require repetitive hand movements at work (14, 22, 23).

PMA and BMN are also common anatomical variations in the wrist, with BMN often coexisting with PMA (10), and BMN has also been reported to coexist with abnormal muscles (24). However, the coexistence of anomalous muscle, PMA, and BMN causing CTS is really rare. Previous studies show that the prevalence of BMN in CTS is about 18%–18.5%, while the prevalence in normal control is approximately 9.4%–15.4%, they believed that BMN was not a rare variant and did not support its etiological relationship with CTS (10, 25). However, Park et al. (26) demonstrated that CTS with BMN showed more severe symptoms and relatively mild electrophysiological diagnosis.

PMA was found in 1.2% to 2.6% of patients during carpal tunnel release surgery, and as high as 8.6% to 23%% at autopsy (27–29). Since the detection rate of PMA in CTS surgery patients is much lower than that in autopsy, the contribution of normal PMA to CTS is questionable. In addition, Altinkaya et al. (30) indicated that the detection rate of PMA in CTS patients was not statistically different from that in normal controls, suggesting that PMA would not increase the risk of CTS. However, thrombosis of PMA commonly leads to the occurrence of acute CTS (31), although the probability of thrombosis in PMA is rare, we suggest that the presence of PMA increases the risk of developing CTS.

In our case, abnormal muscles were present in the bilateral carpal tunnel, and the aberrant muscles were confirmed to be derived from the first lumbrical muscle. The origin of the lumbrical muscle was higher than the normal position, which reduced the volume of the carpal tunnel and caused the median nerve entrapment. However, this patient had no typical paresthesias but had marked thenar muscle atrophy, which we believed was caused by entrapment of the recurrent thenar branch of the median nerve. Interestingly, in addition to the abnormal muscles, the PMA was found in the left carpal tunnel, which divided the median nerve into two branches and caused compression of the median nerve, however, the patient's left hand had milder symptoms than the right. We did not remove the abnormal muscles and PMA during the operation, which may lead to the recurrence of carpal tunnel syndrome in the future, requiring further observation and follow-up.

CTS is very rare in children and adolescents. Differential diagnosis should pay attention to lysosomal storage disease, wrist trauma, carpal tunnel space-occupying lesions, etc., which are more common in children and adolescents with CTS (2, 32). In addition, we thought that it should be differentiated from Congenital thenar hypoplasia and Hirayama disease, especially when only thenar atrophy and weakness are present.

Congenital thenar hypoplasia, also called Cavanagh's syndrome, is a developmental rather than an acquired thenar deformity, which presents with unilateral or bilateral marked flattening and weakness of the thenar eminence, and without paresthesia, pain, numbness of median nerve innervation area (33). Hand radiograph showed typical hypodevelopment of the thumb phalanges and adjacent carpal bones, and the electrophysiologic findings are typical median low-amplitude CMAP, normal latency, conduction velocity, and SNAP (34). Our case has similar clinical manifestations, radiography (Supplementary Figure S3), and electrophysiological findings as above mentioned. However, what distinguishes him from the above description is that MRI and high-resolution ultrasonography found anomalous muscles, PMA, and BMN anatomical variations in the carpal tunnel, and intraoperative exploration confirmed bilateral median nerve compression with altered neural properties. No reports of Cavanagh's syndrome with anatomical variations in the carpal tunnel have been found in the literature. Notably, Cavanagh's syndrome may be co-morbid CTS, and electrophysiologic evaluation of the first lumbrical may assist with the diagnosis (35). Therefore, our diagnosis favors CTS, and possibly with Cavanagh's syndrome.

Hirayama disease (HD), also known as juvenile muscular atrophy of the distal upper extremity, is a rare benign self-limited lower motor neuron disease, mainly involving the hand and forearm, with progressive exacerbation of muscle weakness and muscular atrophy, and MRI of the cervical spine can reveal characteristic imaging changes (36). The patient was misdiagnosed with Hirayama disease before coming to our clinic, however, except for atrophy and weakness of the bilateral thenar, no abnormal cervical spine MRI was found in this patient, so Hirayama disease was ruled out.

Currently, there is no consensus on the management of CTS caused by anatomical abnormalities in the carpal tunnel. For abnormal muscles, some scholars (37) suggested the abnormal muscles should be removed while releasing the median nerve of the carpal tunnel, however, some other authors (14, 15, 18) believed that simply incising the transverse carpal ligament to release the median nerve has achieved the purpose, and there is no need to remove the abnormal muscles. PMA is sometimes a part of the superficial palmar arch, when a thrombus forms in the PMA, conservative treatment with anticoagulation, thrombolysis, or surgical removal of the thrombus can be performed, however, if the PMA does not entrap the median nerve, no treatment is required (3, 28, 31). During the operation, the bilateral flexor retinaculum was incised and the median nerves were released, while the anomalous muscles and the PMA were not removed but were left in place. We believed that the median nerve should be fully released during the operation, and whether to remove the abnormal muscle depends on the intraoperative findings. If median nerve compression persists after sufficient release, resection of the abnormal muscle should be considered.

## Conclusion

Anatomical variations in the carpal tunnel can lead to reduced carpal tunnel volume and contribute to CTS. When CTS occurs in adolescents, the possibility of anatomical variations in the carpal tunnel should be considered, which can be confirmed by preoperative ultrasonography and MRI and can help prevent iatrogenic injury. Open carpal tunnel release is an effective method for the treatment of CTS in adolescents, and it is not necessary to remove the abnormal muscle and PMA during the operation.

## Data Availability

The original contributions presented in the study are included in the article/**Supplementary Material**, further inquiries can be directed to the corresponding author/s.
