# Supplementary figures and images for: Coexistence of anomalous muscle, persistent median artery, bifid median nerve causing carpal tunnel syndrome: A case report and literature review
Source: Front Pediatr. 2023 Feb 9;11:1043442. doi: 10.3389/fped.2023.1043442 (PMC9947498; doi:10.3389/fped.2023.1043442)

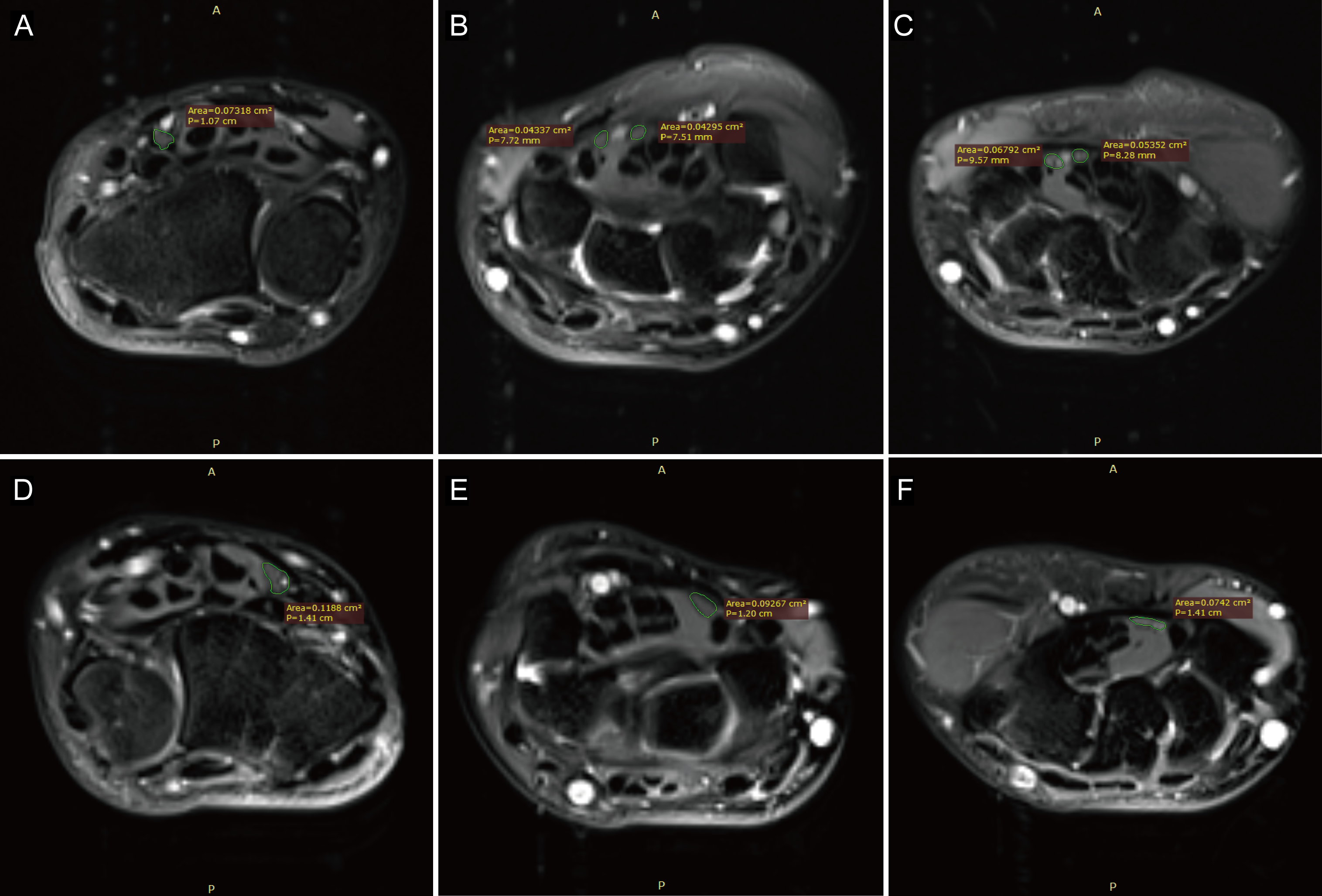

Supplement: Supplementary file 1 [file Image1.tif]

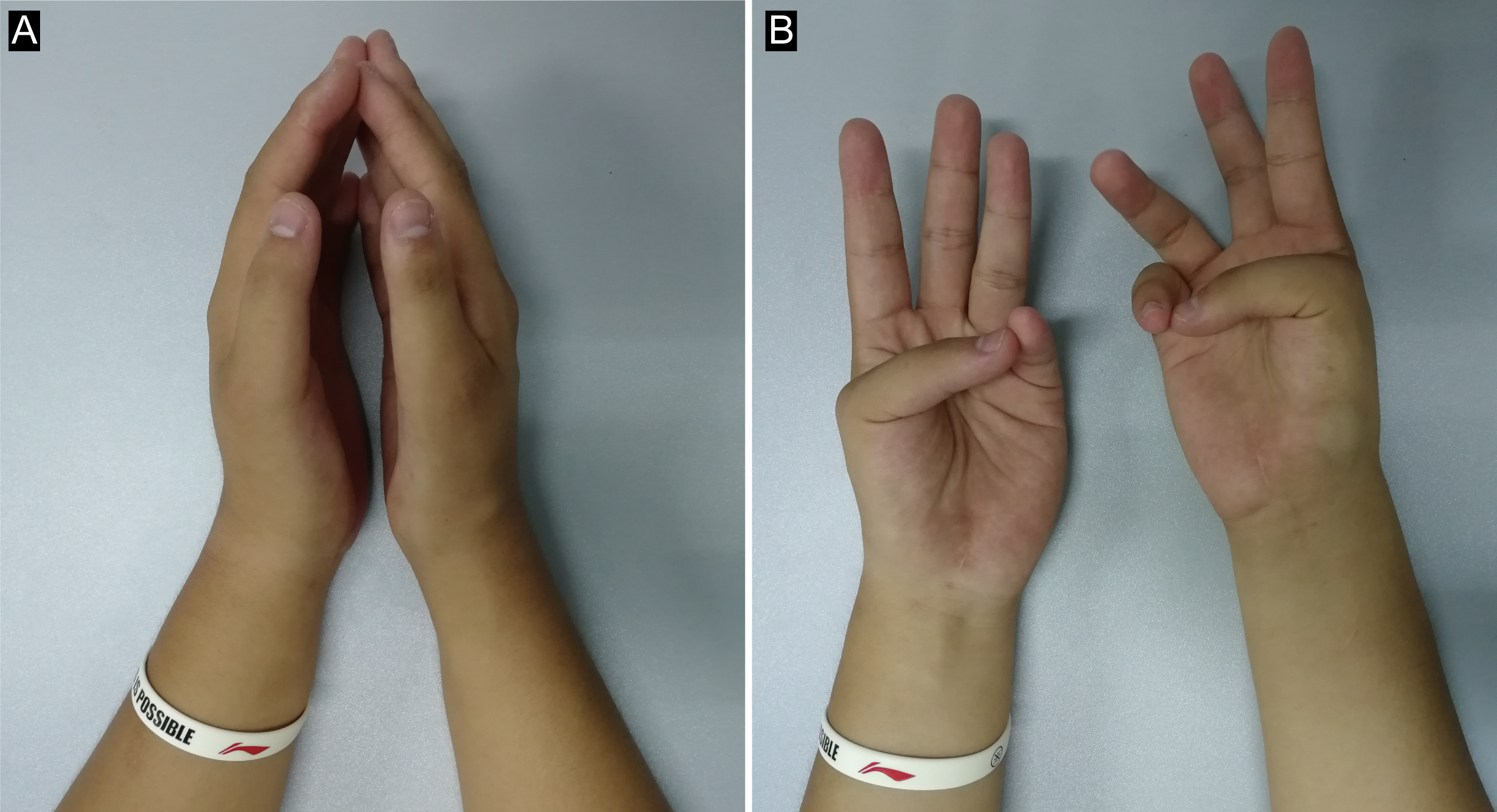

Supplement: Supplementary file 2 [file Image2.tif]

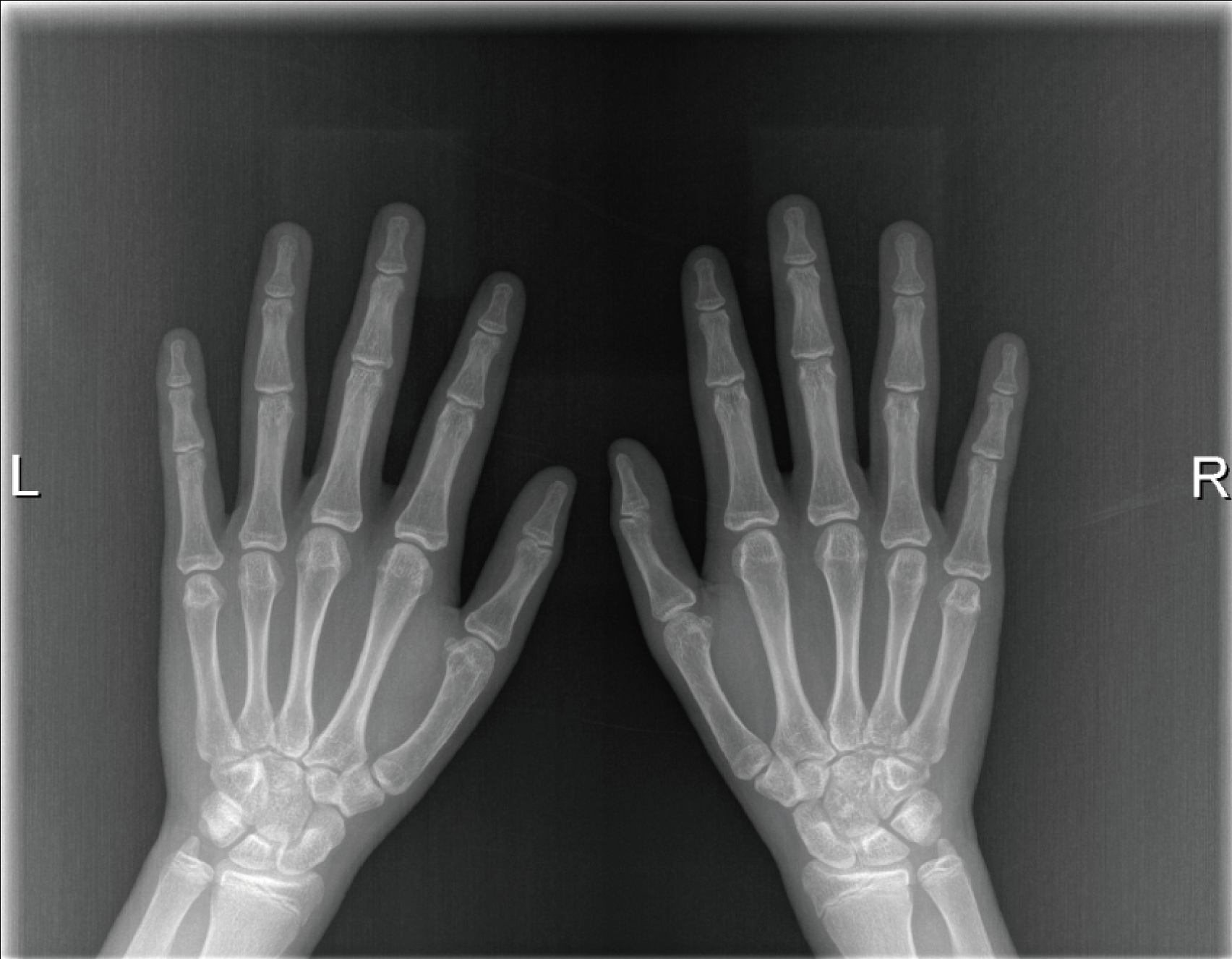

Supplement: Supplementary file 3 [file Image3.tif]
